# Repeat decompression and fusions following posterolateral fusion versus posterior/transforaminal lumbar interbody fusion for lumbar spondylosis: a national database study

**DOI:** 10.1038/s41598-019-41366-z

**Published:** 2019-03-20

**Authors:** Moon Soo Park, Young-Su Ju, Seong-Hwan Moon, Tae-Hwan Kim, Jae Keun Oh, Jin Kyu Lim, Chi Heon Kim, Chun Kee Chung, Ho Guen Chang

**Affiliations:** 1 Department of Orthopaedic Surgery, Hallym University Sacred Heart Hospital, Medical College of Hallym University, 22 Gwanpyeong-ro 170 beon-gi, Dongan-gu, Anyang-si, Gyeonggi-do 14068 Republic of Korea; 2 Department of Occupational and Environmental Medicine, Hallym University Sacred Heart Hospital, Medical College of Hallym University, 22 Gwanpyeong-ro 170 beon-gil, Dongan-gu, Anyang-si, Gyeonggi-do 14068 Republic of Korea; 3 Department of Orthopaedic Surgery, Yonsei University College of Medicine, 50-1 Yonsei-ro, Seodaemun-gu, Seoul, 03722 Republic of Korea; 4 Department of Neurosurgery, Hallym University Sacred Heart Hospital, Medical College of Hallym University, 22 Gwanpyeong-ro 170 beon-gi, Dongan-gu, Anyang-si, Gyeonggi-do 14068 Republic of Korea; 5 Department of Orthopaedic Surgery, Kangdong Sacred Heart Hospital, Medical College of Hallym University, 150, Seongan-ro, Gangdong-gu, Seoul, 05355 Republic of Korea; 6 Department of Neurosurgery, Seoul National University Hospital, 101, Daehak-ro, Jongno-gu, Seoul, 03080 Republic of Korea; 7 Department of Neurosurgery, Seoul National University College of Medicine, 103 Daehak-ro, Jongno-gu, Seoul, 03080 Republic of Korea; 8 Neuroscience Research Institute, Seoul National University Medical Research Center, 103 Daehak-ro, Jongno-gu, Seoul, 03080 Republic of Korea; 9 Clinical Research Institute, Seoul National University Hospital, 101, Daehak-ro, Jongno-gu, Seoul, 03080 Republic of Korea; 10 Department of Brain and Cognitive Sciences, Seoul National University, 1 Gwanak-ro, Gwanak-gu, Seoul, 08826 Republic of Korea

## Abstract

There is a low incidence of reoperation after surgery. It is difficult to detect statistical differences between reoperation rates of different lumbar fusion surgeries. National population-based databases provide large, longitudinally followed cohorts that may help overcome this challenge. The purpose is to compare the repeat decompression and fusion rate after surgery for degenerative lumbar diseases according to different surgical fusion procedures based on national population-based databases and elucidate the risk factor for repeat decompression and fusions. The Korean Health Insurance Review & Assessment Service database was used. Patients diagnosed with degenerative lumbar diseases and who underwent single-level fusion surgeries between January 1, 2011, and June 30, 2016, were included. They were divided into two groups based on procedure codes: posterolateral fusion or posterior/transforaminal lumbar interbody fusion. The primary endpoint was repeat decompression and fusion. Age, sex, the presence of diabetes, osteoporosis, associated comorbidities, and hospital types were considered potential confounding factors. The repeat decompression and fusion rate was not different between the patients who underwent posterolateral fusion and those who underwent posterior/transforaminal lumbar interbody fusion. Old age, male sex, and hospital type were noted to be risk factors. The incidence of repeat decompression and fusion was independent on the fusion method.

## Introduction

Posterolateral fusion with decompression (posterolateral fusion) has been advocated for minimal neural complication compared with posterior/transforaminal lumbar interbody fusion with decompression (posterior/transforaminal lumbar interbody fusion) and has the disadvantage of fusion of posterior column alone. On the other hand, posterior/transforaminal lumbar interbody fusion has the disadvantage of neural complications because of retraction of neural structure in the surgical approaches and the benefit of significantly better fusion rate than the posterolateral fusion. Controversies exist about which surgical procedure is better for the clinical outcome of patients^1–3^.

One of the key factors affecting the postoperative clinical outcomes is reoperation. The incidence of reoperation after surgery for the lumbar degenerative disease is relatively low. Therefore, to sufficiently power studies on detecting differences between reoperation rates of different surgical procedures is difficult. National population-based administrative databases provide a large cohort that may help overcome this challenge and a complete follow-up of reoperations without the follow-up loss, even after the patients are discharged from the hospital.

Administrative data have been widely used for evaluating the reoperation rates after the lumbar surgeries of decompression or fusion with decompression^4–9^. However, few studies have been conducted to evaluate the difference in the reoperation rates between different surgical fusion procedures of posterolateral fusion and posterior/transforaminal lumbar interbody fusion based on national population-based databases and to elucidate the risk factor for reoperations.

This study aimed to compare the repeat decompression and fusion rates after surgery for degenerative lumbar diseases at a single level based on different surgical fusion procedures of posterolateral fusion and posterior/transforaminal lumbar interbody fusion in a national population of patients and elucidate the risk factor for repeat decompression and fusions.

## Results

Posterior/transforaminal lumbar interbody fusion was more commonly encountered in our cohort than posterolateral fusion (58.65% and 41.35%, respectively, Table 1). The mean patient age was 61.86 ± 10.97 years; 63.68% were women (Table 1). Age, sex, the presence of diabetes, associated comorbidities, and hospital types were different between the two groups (Table 1).

**Table 1:** The characteristics of the study population.

	All patients	Posterolateral fusion	Posterior/transforaminal lumbar interbody fusion	p
Number (%)	20,606	8,520 (41.35%)	12,086 (58.65%)	
Age (years)				<0.0001*
20–29	204 (0.99%)	97 (1.14%)	107 (0.89%)	
30–39	537 (2.61%)	227 (2.66%)	310 (2.56%)	
40–49	1,810 (8.78%)	792 (9.30%)	1,018 (8.42%)	
50–59	5,294 (25.69%)	2,041 (23.96%)	3,253 (26.92%)	
60–69	7,306 (35.46%)	2,896 (33.99%)	4,410 (36.49%)	
≥70	5,455 (26.47%)	2,467 (28.96%)	2,988 (24.72%)	
Mean age (SD)	61.86 ± 10.97	62.14 ± 11.38	61.67 ± 10.67	**0.0029** ^†^
Sex, female, n	13,122 (63.68%)	5,341 (62.69%)	7,781 (64.38%)	**0.0129**
Diabetes, n	8,562 (41.55%)	3,635 (42.66%)	4,927 (40.77%)	**0.0065**
Osteoporosis, n	170 (0.83%)	73 (0.86%)	97 (0.80%)	0.6717
Comorbidity, n	10,570 (51.30%)	4,573 (53.67%)	5,997 (49.62%)	**<0.0001**
Hospital types				**<0.0001**
Tertiary-referral hospital	5,303 (25.74%)	2,351 (27.59%)	2,952 (24.42%)	
General hospital	6,355 (30.84%)	2,691 (31.58%)	3,664 (30.32%)	
Hospital	8,598 (41.73%)	3,324 (39.01%)	5,274 (43.64%)	
Clinic	350 (1.70%)	154 (1.81%)	196 (1.62%)	

Distribution of age groups was not different between the two groups (*p < 0.0001).

Mean age was not different between the two groups (^†^p < 0.0029).

In the entire follow-up period, 3.15% of the study population underwent repeat decompression and posterior fusions. The cumulative incidence of repeat decompression and posterior fusions at the end of the study period was similar in the patients who underwent posterolateral fusion (3.15%) and in those who underwent posterior/transforaminal lumbar interbody fusion (3.15%) (Table 2).

**Table 2:** Repeat decompression and posterior fusion rates according to the follow-up periods.

		All patients	Posterolateral fusion	Posterior/transforaminal lumbar interbody fusion
<90 days	Operations	20,606	8,520	12,086
	Reoperations	37 (0.18%)	17 (0.20%)	20 (0.17%)
90 days to 4 years	Operations	20,569	8,503	12,066
	Reoperations	548 (2.66%)	231 (2.72%)	317 (2.63%)
≥4 years	Operations	20,021	8,272	11,749
	Reoperations	64 (0.32%)	20 (0.24%)	44 (0.37%)
Total period	Operations	20,606	8,520	12,086
	Reoperations	649 (3.15%)	268 (3.15%)	381 (3.15%)

**Figure 1:**
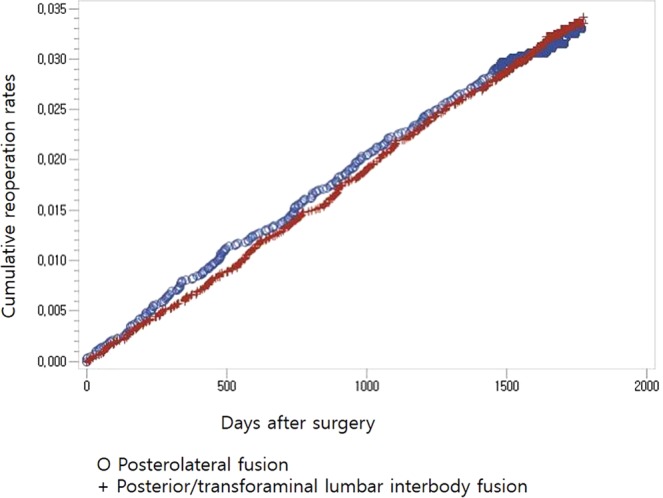
Cumulative repeat decompression and posterior fusion rate of surgical procedures according to surgical procedure during the entire follow-up period.

**Figure 2:**
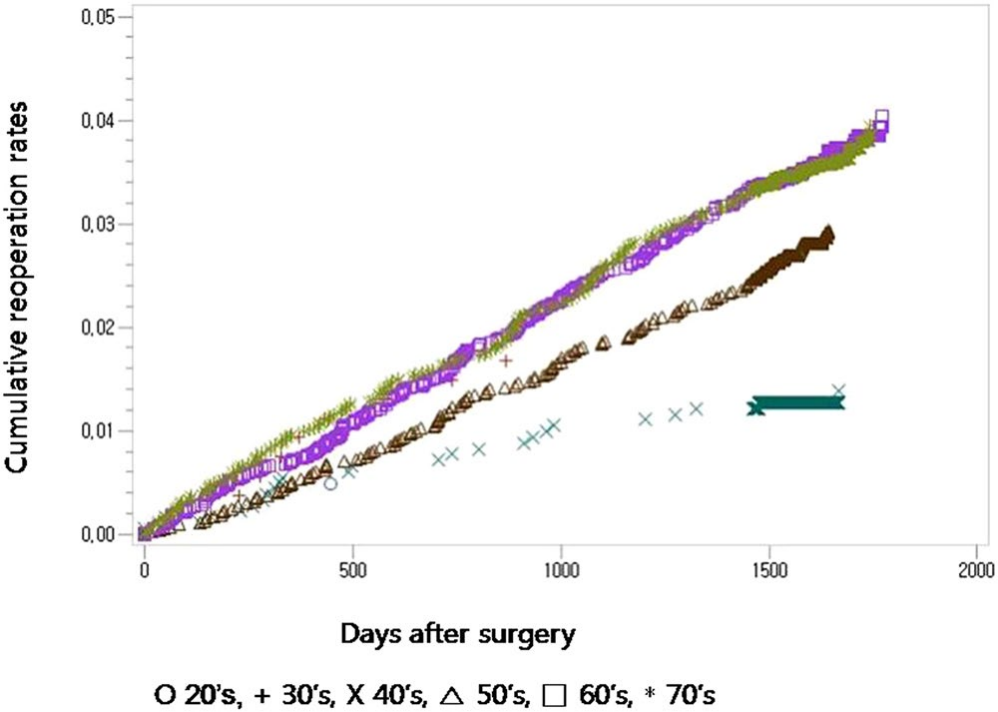
Cumulative repeat decompression and posterior fusion rate of surgical procedures according to age groups during the entire follow-up period.

**Figure 3:**
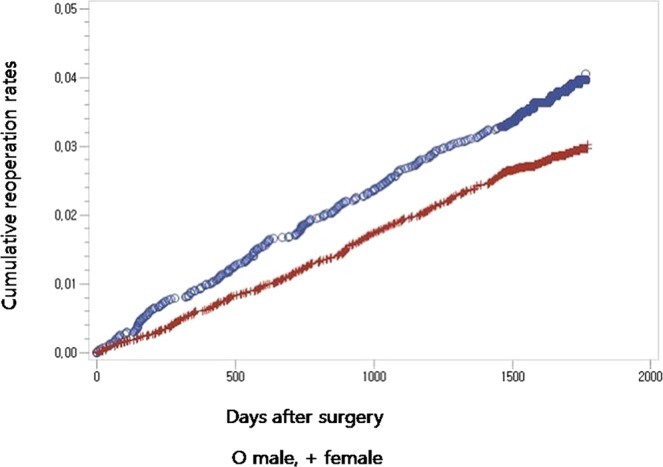
Cumulative repeat decompression and posterior fusion rate of surgical procedures according to sex during the entire follow-up period.

No difference was found in the unadjusted repeat decompression and posterior fusion rate for surgical procedures between the patients who underwent posterolateral fusion and those who underwent posterior/transforaminal lumbar interbody fusion (Fig. 1, Table 3). Age, sex, the presence of diabetes, associated comorbidities, and hospital types were detected to be significant confounding factors by Cox regression analysis (Table 3). After adjusting for these confounders, age, sex, and hospital types were all found to significantly affect the risk for repeat decompression and posterior fusion (patients in their 60 s: p = 0.0310, hazard ratio = 8.681, 95% confidence interval [CI] 1.218–61.860; patients in their 70 s: p = 0.0341, hazard ratio = 8.374, 95% CI 1.173–59.758 (Fig. 2); female sex: p < 0.0001, hazard ratio = 0.725, 95% confidence interval [CI] 0.620–0.848 (Fig. 3); general hospital: p = 0.0099, hazard ratio = 1.309, 95% confidence interval [CI] 1.067–1.606 (Table 4)).

**Table 3:** Comparison between surgical procedures based on the unadjusted value of Cox regression analysis.

	Entire period (n = 20,606)		
	p	Hazard ratio	95% Confidence interval
**Surgical procedures**			
Posterolateral fusion		1.000	
Posterior/transforaminal lumbar interbody fusion	0.9559	1.004	(0.859, 1.174)
**Age (years)**			
20–29		1.000	
30–39	0.2415	3.433	(0.436, 27.057)
40–49	0.3284	2.709	(0.367, 19.994)
50–59	0.0793	5.808	(0.814, 41.432)
60–69	**0.0426**	7.615	(1.071, 54.165)
≥70	**0.0437**	7.543	(1.059, 53.725)
**Sex**			
Male		1.000	
Female	**0.0005**	0.757	(0.648, 0.885)
**Diabetes**			
Yes	**0.0005**	1.317	(1.129, 1.537)
No		1.000	
**Osteoporosis**			
Yes	0.4583	1.325	(0.630, 2.791)
No		1.000	
**Comorbidities**			
Yes	**<0.0001**	1.384	(1.184, 1.619)
No		1.000	
**Hospital types**			
Tertiary-referral hospital		1.000	
General hospital	**0.0051**	1.336	(1.091, 1.637)
Hospital	0.8747	0.984	(0.803, 1.205)
Clinic	0.7890	1.087	(0.590, 2.005)

**Table 4:** Comparison between surgical procedures based on the adjusted value of Cox proportional hazards regression modeling.

	Entire period (n = 20,606)		
	p	Hazard ratio	95% Confidence interval
**Age (years)**			
20–29		1.000	
30–39	0.2249	3.591	(0.456, 28.304)
40–49	0.2776	3.027	(0.410, 22.360)
50–59	0.0581	6.696	(0.937, 47.841)
60–69	**0.0310**	8.681	(1.218, 61.860)
≥70	**0.0341**	8.374	(1.173, 59.758)
**Sex**			
Male		1.000	
Female	**<0.0001**	0.725	(0.620, 0.848)
Diabetes			
Yes	0.7018	1.052	(0.812, 1.362)
No		1.000	
**Comorbidities**			
Yes	0.1901	1.194	(0.916, 1.556)
No		1.000	
**Hospital types**			
Tertiary-referral hospital		1.000	
General hospital	**0.0099**	1.309	(1.067, 1.606)
Hospital	0.9552	0.994	(0.811, 1.218)
Clinic	0.6883	1.134	(0.614, 2.092)

## Discussion

National population-based databases provide complete information about reoperations without the follow-up loss because the patients had the reoperations in the hospitals in Korea, even after they discharged from the initial hospital after the index operation. The claim-based approach for reporting reoperation was comparably accurate compared with medical records abstraction because the fee-for-service reimbursement system in Korea requires procedure codes of service for every surgical fee^10^. No study has been conducted to evaluate the difference in the reoperation rates between the different lumbar surgical fusion procedures subgroups with the national population database. Therefore, this study aimed to compare the repeat decompression and posterior fusion rates after surgery for degenerative lumbar diseases according to different surgical fusion procedures of posterolateral fusion and posterior/transforaminal lumbar interbody fusion at single disc level in a national population of patients and elucidate the risk factor for repeat decompression and posterior fusions.

The repeat decompression and posterior fusion rate was not different between the patients with posterolateral fusion and those with posterior/transforaminal lumbar interbody fusion. Old age, male sex, and hospital type were noted to be risk factors for repeat decompression and posterior fusion.

The repeat decompression and posterior fusion rates were similar between the two groups. It might be explained by the fact that the reoperations are required because of nonunion and other complications besides nonunion. The posterior/transforaminal lumbar interbody fusion leads to better fusion rates than posterolateral fusion because posterior/transforaminal lumbar interbody fusion fuses the anterior column and posterior column^2,3,11^. However, the complication rates were higher in the patients who underwent posterior/transforaminal lumbar interbody fusion than those who underwent posterolateral fusion in the study based on the administrative database with the follow-up of 10 years^12^.

Campbell *et al*.^13^ made a comparison between the reoperation rates after lumbar surgeries of posterolateral fusion and those after posterior lumbar interbody fusion for the degenerative spondylolisthesis using meta-analysis based on six studies. They found no difference in the reoperation rate between the patients who underwent posterolateral fusion and those who underwent posterior lumbar interbody fusion. Similarly, Luo *et al*.^14^ evaluated the reoperation rates after lumbar surgeries for isthmic spondylolisthesis using meta-analysis based on nine studies. They reached the same conclusion. Both studies are concordant with the current study.

Our study does not elucidate the reason why patients with risk factors proven in the current study might have higher repeat decompression and posterior fusion rates. Possible explanations are given below.

Old age affects the risk of repeat decompression and posterior fusion in the current study. The similar conclusion has also been made in the studies on the reoperation rate after cervical fusion for cervical degenerative diseases^15,16^, and after lumbar surgeries for disc herniation^8^. Male sex affects the risk of repeat decompression and posterior fusion in the current study. However, the reason was unknown. A possible explanation is that male sex was associated with the reoperations due to adjacent segmental disease in the retrospective clinical study with 163 patients who underwent decompressive surgeries or posterior lumbar interbody fusion surgeries for the degenerative spondylolisthesis^17^. The hospital type of general hospital was a risk factor for repeat decompression and posterior fusion in the current study. It might be due to that tertiary-referral hospitals were better equipped with a variety of diagnostic tools and full of essential departments than the general hospital does.

As with any study, our investigation has several limitations. First, clinical information about pain, quality of life, and functional and neurologic status were not available in the administrative dataset. Radiographic information, level of complexity of surgeries, the information about smoking/nicotine dependence and bone graft material were also not available. The clinical and radiologic information of the patients about the surgical indications was not available. Fortunately, all hospitals in Korea follow the requirements of surgical treatments from the Korean National Health Insurance Corporation for reimbursement. The requirements include the symptoms, neurologic and radiologic findings of patients for their surgeries, which are considered as the surgical indications. Adjacent segment disease requiring reoperation is different from pseudoarthrosis at the index level requiring reoperation. These two different indications could not be separated in the analysis of the current study. In addition, no information was available about how many patients who were indicated for revision surgery had refused. Therefore, the exact reasons for repeat decompression and posterior fusions were not available. These restrictions are inherent to administrative databases^18^. However, the large sample size of our cohort allowed for the estimation of average reoperation rates that are generalizable to the entire population. Second, in the current study, the primary endpoint of reoperation was a repeat posterior fusion surgery. Therefore, other reoperations such as discectomies, laminectomies, incision and drainage for surgical wound infection, and anterior fusion surgeries were not evaluated. The reoperation rates may be underestimated because the reoperations were limited to posterior fusion surgeries. Another limitation is that this was a population study of Koreans operated on by Korean surgeons. Therefore, it may not be generalizable to other countries or regions. However, the Korean healthcare system uses modern and the latest surgical techniques that are not appreciably different than those utilized in most first-world countries. Finally, We have analyzed the data, combining the patients who underwent posterior lumbar interbody fusion and those who underwent transforaminal lumbar interbody fusion techniques in the current study. It is attributable to the technical problem that the procedure code for posterior lumbar interbody fusion and a procedure code for transforaminal lumbar interbody fusion were the same in the Korean Health Insurance Review & Assessment Service (HIRA) database. Fortunately, clinical outcomes were not different between the patients who underwent posterior lumbar interbody fusion and those who underwent transforaminal lumbar interbody fusion^19–22^, even though the perioperative complications including wound infection, hematoma, neurologic deficit, cerebrospinal fluid leakage, and screw misplacement was higher in the patients who underwent posterior lumbar interbody fusion^21–23^. There has been no study comparing the long-term complication of the repeat decompression and posterior fusion rates between the two groups. In future, we have plan to compare the repeat decompression and posterior fusion rates between the two groups. Despite these limitations, based on our literature review, this study represents the first population-based analysis of the repeat decompression and posterior fusion rates, comparing the patients who underwent posterolateral fusion and those who underwent posterior/transforaminal lumbar interbody fusion.

In conclusion, the repeat decompression and posterior fusion rate was not different between the patients with posterolateral fusion and those with posterior/transforaminal lumbar interbody fusion at a single disc level. This finding could help surgeons to more accurately communicate risks of surgery to patients. Therefore, all patients can make fully educated decisions about whether to undergo surgery.

## Material and Methods

This study was approved by the institutional review board of Hallym University Sacred Heart Hospital (IRB number: 2016-I106). The institutional review board waived the informed consent for this study.

### Data source

The Korean Health Insurance Review & Assessment Service (HIRA) database is a national, prospectively collected set of data that includes roughly 51 million patients enrolled in the Republic of Korea. It contains all inpatient and outpatient data reported by diagnosis and procedure codes. The diagnosis codes are standardized according to the Korean Classification of Disease, 6th version, which follows the International Classification of Disease, 10th edition (ICD-10).

### Study population selection and design

The HIRA national database was searched to identify patients who had a primary diagnosis of lumbar disc herniation (diagnosis codes: M4720, M4721, M4722, M4723, M4724, M4725, M4726, M4727, M4728,M4729, M5410, M5412, M5413, M5419, M511), degenerative spondylolisthesis (diagnosis codes: M4310, M4311, M4312, M4313, M4314, M4315, M4316, M4317, M4318, M4319), or degenerative spinal stenosis (diagnosis codes: M4800, M4801, M4802, M4803, M4804, M4805, M4806, M4807, M4808, M4809, M9920, M9921, M9922, M9923, M9924, M9951, M9952, M9953, M9954). The subjects were included if they had any of the following primary procedures of posterior fusion combined with the procedure of posterior decompression between January 1, 2011, and June 30, 2016: first, lumbar posterolateral fusion (procedure code: N0469, N1469) with lumbar laminectomy (procedure code: N1499, N2499) or lumbar discectomy (procedure code: N1493), and second, posterior/transforaminal lumbar interbody fusion (procedure code: N2470, N1460) with lumbar laminectomy (procedure code: N1499, N2499) or lumbar discectomy (procedure code: N14930). The patients’ resident registration numbers were encrypted for privacy. We have adopted the study design of the current study from previous studies because it is most effective to elucidate the reoperation rate after surgeries^8,24^.

**Figure 4:**
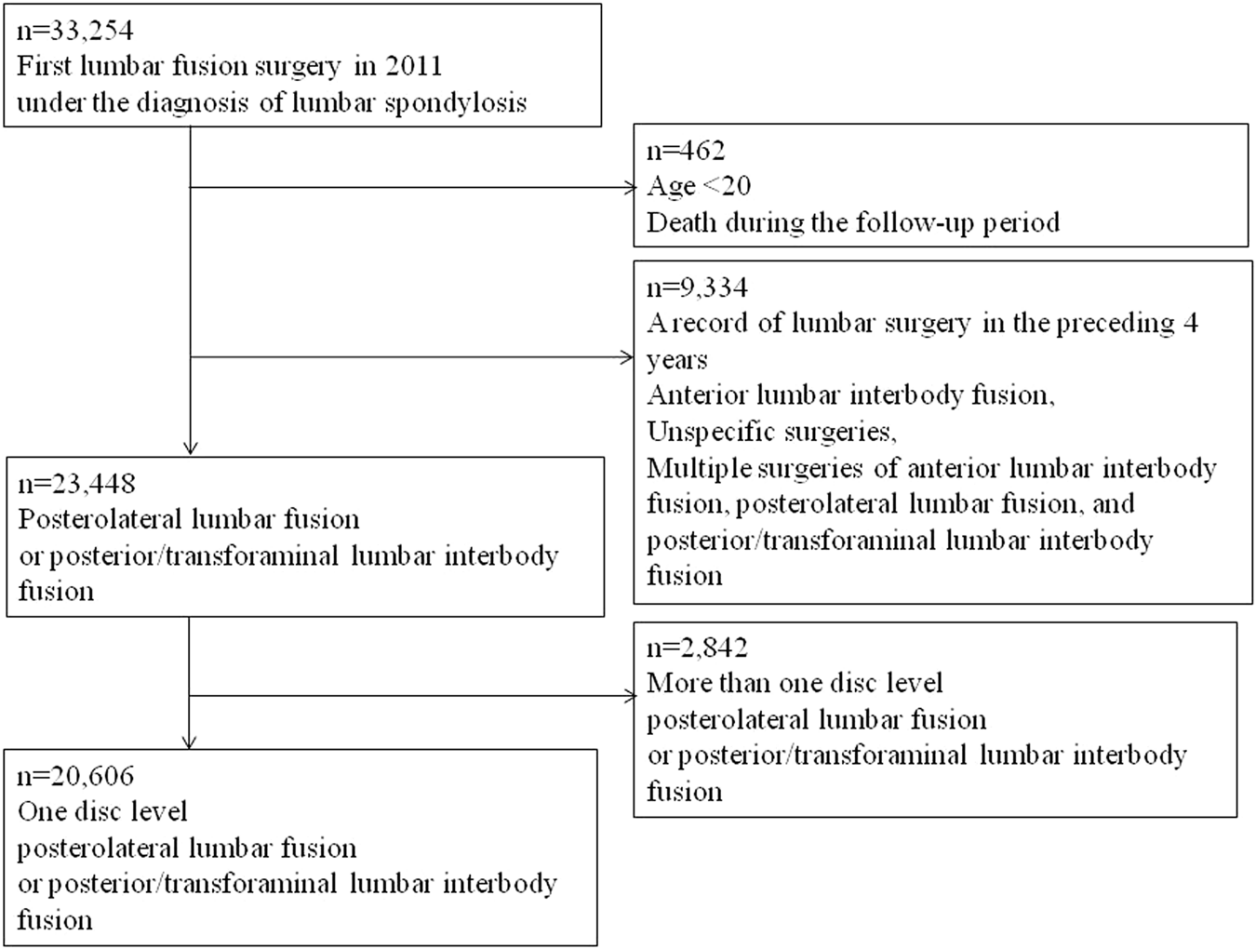
Cohort definition.

A total of 33,254 patients who underwent lumbar fusion surgery under the diagnosis of lumbar spondylosis in 2011 were identified from the cohort of patients (Fig. 4). The patients who were aged <20 years and those who had died during the follow-up period (causes of death were not recorded) were then excluded. They were excluded if they had a history of lumbar surgery within the preceding 4 years (2007–2010), underwent anterior lumbar interbody fusion, or had undergone multiple of the above procedures or lumbar surgeries that were not specified. Additionally, the patients were excluded from the study if they underwent posterolateral lumbar fusion or posterior/transforaminal lumbar interbody fusion at >1 disc level. The final study population of the patients who underwent posterolateral lumbar fusion or posterior/transforaminal lumbar interbody fusion at a single disc level in 2011 was 20,606 patients.

All patients included in the study cohort were evaluated for the follow-up period of four and half years between January 1, 2012, and June 30, 2016.

The subjects were divided into two groups based on the respective procedure codes: posterolateral lumbar fusion or posterior/transforaminal lumbar interbody fusion. Our goal was to determine changes in the repeat decompression and fusion rates over time and to compare the repeat decompression and fusion rates of the abovementioned surgical procedures while adjusting for confounding variables.

### Surgical indications

Nearly all hospitals in Korea follow the requirements of surgical treatments from the Korean National Health Insurance Corporation for reimbursement. In Korea, the standard of surgical care for patients with lumbar radiculopathy is lumbar discectomy in case of patients with intractable pain or neurologic deficit despite nonsurgical treatment for at least 12 weeks. For lumbar fusion of lumbar radiculopathy, these regulations additionally require recurred lumbar disc herniation or foraminal lumbar disc herniation or combination of lumbar instability and lumbar radiculopathy. The standard of surgical care for patients with degenerative spondylolisthesis and spinal stenosis in Korea is posterior lumbar decompression in case of patients with unimproved symptoms despite nonsurgical treatment for at least 12 weeks. For lumbar fusion of degenerative spondylolisthesis and spinal stenosis, these regulations additionally require foraminal stenosis or combination of lumbar instability and degenerative spondylolisthesis or spinal stenosis. Therefore, these Korean National Health Insurance Corporation requirements were considered as the surgical indications for patients in this cohort. There are no regulations for the use of posterolateral lumbar fusion or posterior/transforaminal lumbar interbody fusion in Korea and it depends on the surgeon’s preference.

### Confounding factors

In the current study, age, sex, the presence of diabetes, osteoporosis, medical comorbidities, and hospital types were considered potential confounding factors. Medical comorbidities were assessed according to the “International Classification of Disease, Ninth Edition, Clinical Modification (ICD-9-CM) and ICD-10 coding algorithms for Charlson Comorbidities” proposed by Quan *et al*.^25^ If there were >4 distinct primary or secondary diagnoses in 2009, the patients were regarded as having associated medical comorbidities^6,8^. Diabetes was analyzed separately as it is a known risk factor for reoperation that increases complication rates and inhibits functional recovery^8,26^.

In Korea, hospital types are determined by law^8^. General hospitals have at least seven departments, such as internal medicine, general surgery, obstetrics and gynecology, pediatrics, diagnostic radiology, anesthesiology, pathology, and laboratory medicine. In addition, they also must have at least one board-certified doctor in each department with >99 beds. Tertiary-referral hospitals are distinguished from general hospitals by having at least 20 departments. In addition to the characteristics of general hospitals, tertiary-referral hospitals also have residency training programs, at least 5 operating rooms, and a variety of diagnostic tools, including computed tomography, magnetic resonance imaging, electromyography, angiography, gamma camera radiography, and Holter cardiac monitoring. Hospitals are healthcare systems that do not have essential departments or those that have between 30 and 99 beds. Private clinics have <30 beds.

### Statistical analysis

Time to event (repeat decompression and posterior fusion) survival analysis was performed. The primary endpoint was any repeat of posterior decompression and lumbar fusion during follow-up. Reoperations (repeat decompression and posterior fusions) were identified by the presence of any of the aforementioned primary procedures of a combination of posterior fusion and posterior decompression recorded after the index procedure code. Therefore, reoperation included lumbar operations performed at both the original or different levels. They included the reoperations for revision of the original levels and adjacent segmental diseases at different levels. The third and subsequent reoperation events were excluded from the cumulative operation rates since those later interventions may not have portrayed the natural history after lumbar operations. If the latter date was not available, January 1, 2011, which is the first date in our data collection period, and June 30, 2016, which is the last date, were used. Therefore, the minimal follow-up period is four and a half years (from January 1, 2012, to June 30, 2016). To compare baseline characteristics of the subjects, chi-square tests or analysis of variance was used. Statistical analysis for comparison among the two surgical groups was performed using Cox proportional hazards regression modeling. Data were analyzed using the SAS software version 0.6.1 (SAS Institute, Inc., Cary, NC, USA). A *p* value of <0.05 was considered statistically significant.

## Data Availability

The datasets generated during and/or analyzed during the current study are available from the corresponding author on reasonable request.
